# The neural correlates of picture naming facilitated by auditory repetition

**DOI:** 10.1186/1471-2202-13-21

**Published:** 2012-02-27

**Authors:** Shiree Heath, Katie McMahon, Lyndsey Nickels, Anthony Angwin, Anna MacDonald, Sophia van Hees, Kori Johnson, David Copland

**Affiliations:** 1 University of Queensland, Language Neuroscience Laboratory, Centre for Clinical Research, Brisbane, Queensland, Australia; 2 University of Queensland, Centre for Advanced Imaging, St Lucia, Queensland, Australia; 3 ARC Centre of Excellence in Cognition and its Disorders, Department of Cognitive Science, Macquarie University, Sydney, New South Wales, Australia; 4 University of Queensland, School of Health and Rehabilitation Sciences, St Lucia, Queensland, Australia

## Abstract

**Background:**

Overt repetition of auditorily presented words can facilitate picture naming performance in both unimpaired speakers and individuals with word retrieval difficulties, but the underlying neurocognitive mechanisms and longevity of such effects remain unclear. This study used functional magnetic resonance imaging to examine whether different neurological mechanisms underlie short-term (within minutes) and long-term (within days) facilitation effects from an auditory repetition task in healthy older adults.

**Results:**

The behavioral results showed that both short- and long-term facilitated items were named significantly faster than unfacilitated items, with short-term items significantly faster than long-term items. Neuroimaging analyses identified a repetition suppression effect for long-term facilitated items, relative to short-term facilitated and unfacilitated items, in regions known to be associated with both semantic and phonological processing. A repetition suppression effect was also observed for short-term facilitated items when compared to unfacilitated items in a region of the inferior temporal lobe linked to semantic processing and object recognition, and a repetition enhancement effect when compared to long-term facilitated items in a posterior superior temporal region associated with phonological processing.

**Conclusions:**

These findings suggest that different neurocognitive mechanisms underlie short- and long-term facilitation of picture naming by an auditory repetition task, reflecting both phonological and semantic processing. More specifically, the brain areas engaged were consistent with the view that long-term facilitation may be driven by a strengthening of semantic-phonological connections. Short-term facilitation, however, appears to result in more efficient semantic processing and/or object recognition, possibly in conjunction with active recognition of the phonological form.

## Background

Word retrieval is often targeted clinically in the treatment of individuals suffering from the naming difficulties associated with post-stroke aphasia. One common form of word retrieval treatment involves repeating a target name in the presence of the target picture. This task is often framed as a phonological treatment and is assumed by some to improve word retrieval by targeting phonological representations. However, such a task may also improve word retrieval by increased semantic activation or strengthening of mappings between semantics and phonology [1]. The neural mechanisms underpinning such effects are not well known in either healthy individuals or those with aphasia. It has been shown that certain aspects of language recovery in aphasia may involve regions also recruited in healthy individuals, for instance during lexical learning [2]. In fact, it has been proposed that normal priming mechanisms may underlie the successful treatment of word retrieval in aphasia [3]. A better understanding of these priming mechanisms in unimpaired speakers could aid development of more theoretically driven and neurobiologically informed treatment methods. Therefore, the present study used functional magnetic resonance imaging (fMRI) to investigate in healthy older adults the effects associated with a commonly used treatment technique on subsequent picture naming performance.

The spoken production of a picture name is a complex linguistic operation, requiring integration of perceptual, semantic, phonological and articulatory processes. Thus the ability to name an object involves multiple, functionally separable, sub-processes. During the semantic stage, successful word production requires the meaning of a picture to be activated within the semantic system, which is a store of word meanings [4]. Conceptual representations must then be translated into word-level knowledge, by selection of the lexical entry that matches the picture representation. This abstract lexical unit is given phonetic form during the phonological stage [4], where the phonological properties of the word are brought together for articulation. A mapping operation must also exist between the semantic and phonological systems, linking word meaning and word form [5]. Ease of access to the phonological level from the semantic system relies on the strength of these links [6,7]. These three component processes of picture naming (i.e., semantic, phonological and connections between) represent the basic architecture of the lexical system shared by most theoretical models of word production. Word production is supported by a network of perisylvian neural regions involving the frontal, parietal and temporal lobes. It also appears that the semantic and phonological components of single word production engage different regions [8]. The anterior and mid-portions of the inferior frontal gyrus, the middle and inferior temporal gyri, and the angular gyrus of the parietal lobe have been associated with semantic processing [9-15]. Phonological processing, however, has implicated the posterior portion of the inferior frontal gyrus (but see [16]), the superior temporal gyrus and the supramarginal gyrus of the parietal lobe [8,9,11,14,17,18].

Importantly, our ability to successfully activate, select and produce a specific name can be positively influenced by certain factors. By way of example, two of these intrinsic factors include frequency and age of acquisition. Word frequency refers to the number of times a particular word occurs in spoken or written English and pictures that have names occurring more frequently are named faster than those occurring less frequently [19,20]. The age at which a word was learnt by an individual also influences picture naming latencies, with pictures associated with earlier acquired words recognized and produced faster than later acquired items [21,22] and this effect appears to persist well into older age [23]. Certain intrinsic properties of words can influence naming performance, however, it is also widely recognized that the simple act of naming a picture once, speeds subsequent naming of that picture [24,25]. This performance enhancement is referred to as priming. A variety of tasks have been used to prime picture naming, including prior instances of phonological processing related to the picture.

Contrasting views exist in the behavioral literature, however, regarding the locus of priming using an auditory repetition task. It has been suggested that this is a phonologically-based task, which facilitates subsequent naming by priming the word form representation, made available during the phonological stage [19]. Others have proposed that phonological tasks facilitate naming by strengthening the connections between the semantic and phonological levels of processing [26]. Adding further complexity to this issue, facilitation techniques targeting different components of the naming process appear to have different time courses. It has been proposed that facilitation at the phonological level of processing results in only short-term benefits, while a strengthening of semantic-phonological connections is associated with longer lasting facilitation. Behavioral evidence for this suggestion was provided by Wheeldon and Monsell [26] who investigated the effects on picture naming of previously producing the name of an item in response to a definition over the short-term (10 to 35 s) or long-term (6 to 12 min). Their results identified strong facilitation over both time frames. They stated that facilitation must have brought about a change in the cognitive pathway shared by both producing a name in response to a definition and naming a picture. In contrast, a second experiment by the same authors found no facilitation of picture naming (6 to 12 min later) from previous production of a homophone of the target item (a word that shares pronunciation, but does not share meaning, e.g. flour, flower). Wheeldon and Monsell [26] concluded that repeated production of the phonological word form was not sufficient to produce priming effects over long lags. They, therefore, attributed repetition priming effects over a period of minutes to a strengthening of the connections between semantics and phonology, rather than to changes in accessibility of the phonological representation itself. Implicit memory research generally supports this view by attributing short-term repetition priming effects to a heightened accessibility of lexical representations lasting only a few seconds, whereas longer-lived priming is accounted for by more explicit episodic memory mechanisms [27,28].

A number of neuroimaging studies have investigated the neural regions engaged by the different component processes of word production by manipulating the phonological or semantic processing involved, often within a repetition priming context. Relevant to the present study is the body of research attempting to identify the brain regions selectively engaged by phonological processing. This research has employed pictures, real words and nonwords in a variety of visual and auditory tasks, including picture naming [29-32], word repetition [26,33], word reading [29,34,35], word generation [9,36], word stem completion [37], word interference [38], rhyming [39,40], and phoneme or syllable discrimination tasks [12,39,41]. Typically these studies, as well as several language-related reviews and meta-analyses [8,13,14,42], have identified facilitation of subsequent responses associated with a decrease of neural activity in various regions, such as portions of the inferior frontal gyrus, the superior temporal gyrus and the supramarginal gyrus of the parietal lobe. It is evident though, that most research has either simply contrasted tasks involving phonological or semantic processing, compared areas of neural activation engaged by semantic or phonological language tasks relative to some sort of baseline activity [42], or used the same task on prime and target presentations [43-45]. Few studies have explored repetition priming effects using a specific facilitatory prime task directed at one of the component processes of naming to investigate the longevity of any subsequent effects. In this regard, the majority of repetition priming research investigates effects over short periods, often less than 30 s. The present study considers the facilitation effects of a phonological task over a period of several minutes (in the short-term), and over a period of days (in the long-term) which may be indicative of a stable and more enduring change in processing [31,46].

Two neuroimaging studies have explored the neural mechanisms mediating very long-lasting facilitation of picture naming. In a study by van Turennout et al. [30] a picture naming task was used at different time intervals prior to scanning and during a subsequent fMRI scanning session up to 3 days later. Subjects were asked to name objects aloud during prime presentations, but during the scanning session were required to silently name each item, such that task compliance and reaction times could not be measured. A decrease of activity for multiple exposures to stimuli was found in bilateral occipitotemporal regions and left inferior frontal cortices, as well as an increase in activity in the left anterior insula and left basal ganglia [30]. This activity was time dependent in the inferior frontal cortex, with larger decreases in activity when more time intervened between successive exposures [30]. The authors suggest this finding is consistent with experience-related changes in activity, resulting in less effort being required to encode and identify a repeated object name [47] in posterior regions and the existence of a procedural learning mechanism in more anterior regions, as well as the basal ganglia and insula cortex [30].

Meister et al. [31] also looked at the priming of very long-term picture naming, in this case up to 6 weeks after initial exposure. An overt naming task was employed in pre-scan sessions and a covert naming task was employed during scanning. Behavioral picture priming effects were associated with reduced activity in the posterior inferior temporal cortex and the anterior inferior frontal region for both short-term (one day) and long-term (6 weeks) intervals [31]. Although both studies found priming-related mechanisms in neural regions associated with naming over a period of weeks [30,31], it is difficult to determine which components of the word production process are contributing to these long lasting priming effects. This is due to the fact that any one, or combination, of the different levels of word production could be contributing to the neural changes resulting from repetition priming when a picture naming task is used at prime presentation and during scanning. The present experiment builds upon this previous work in a significant way by requiring participants to produce overt picture naming responses within the fMRI scanning session and, importantly, by utilizing a different task on prime presentation. An auditory repetition task was used to target the phonological component of naming, and allowed investigation of the longevity of any facilitation effects upon subsequent picture naming which may arise from this specific aspect of word production over a period of days.

In an effort to build upon previous research and discriminate between competing claims regarding the basis and longevity of repetition priming, the present study used fMRI to investigate the neurocognitive substrates underlying facilitation of word retrieval by a phonological technique. The auditory repetition task utilized in this study was performed in the presence of a picture and was used to facilitate subsequent overt naming of the same picture over long (within days) and short (within minutes) time frames. Importantly, the three main naming conditions of interest were presented in a single scanning session. It was hypothesized that any short-term facilitation effects should primarily engage regions associated with phonological processing, indicative of the fact that temporary facilitation may be occurring at the phonological level of processing. On the basis of previous neuroimaging research [8,13-15,42] we expected these areas to include the posterior portions of the inferior frontal and superior temporal gyri, and the supramarginal gyrus of the parietal lobe. Additionally, we hypothesized that long-term facilitation could involve brain areas linked to both semantic and phonological processing, which would suggest that the longevity of facilitation from a phonological task relies on a strengthening of the connections between semantic and phonological levels of processing. These semantic regions, in addition to areas associated with phonological processing, include the anterior portion of the inferior frontal gyrus, the middle and inferior temporal gyri, the angular gyrus of the parietal lobe, and possibly regions linked to episodic memory [8,13-15,42].

## Methods

### Participants

Twenty-one (12 female) healthy older adults (average age 56.9, SD = 9.5, range 38 to 74 years) were recruited to participate in the study. The average educational level of participants was 16.3 years (SD = 4.1, range 10 to 25 years). Participants received no direct financial benefit, but were reimbursed for travel costs. All were right handed, had normal or corrected to normal vision and were native speakers of English. Exclusionary criteria included significant hearing loss (identified by pure tone audiometry screening), any neurological disease or disorder, mental illness, a history of alcohol abuse, as well as the presence of any metal objects within the body, or other contraindications for magnetic resonance imaging. In addition, participants were tested for visual acuity, screened for cognitive impairment with the Mini-Mental State Examination [48] and for depression with the Geriatric Depression Scale [49]. Full ethical approval was obtained from the University of Queensland Medical Research Ethics Committee and written informed consent obtained from each participant in accordance with the Declaration of Helsinki.

### Stimuli

The 60 experimental stimuli and 12 practice stimuli were sourced from the Hemera digital photographic database (Hemera Photo-Objects, Hemera, Hull, Canada) and other royalty-free digital stock photographs across ten broad semantic categories (including objects, animals, food, clothing, people, vehicles, tools, places, natural phenomena, and body/animal parts). All images were photographs of approximately the same size (no larger than 500 × 420 pixels) with a consistent white background canvas size (600 × 600 pixels). The images were grey-scaled with an average luminance of 223.68 candela per m^2 ^(SD = 19.84, range 151.44 to 253.67). Mean reaction times and percentage name agreement data was obtained from the International Picture Naming Project database [50]. Frequency counts were sourced from the CELEX lexical database [51] and age of acquisition norms from Morrison et al. [21]. Associated imageability ratings were obtained from the Medical Research Council psycholinguistic database [52]. The 60 stimuli were divided into three sets of 20 items, with each set assigned to a different main condition of interest - unfacilitated, short-term facilitated or long-term facilitated. Assignment of sets to conditions was counterbalanced across participants. Critical sets were matched (*p *< 0.05) on the basis of reaction time (mean 926.66, SD 173.30, range 656 to 1452), frequency (mean 3.04, SD 1.29, range 0.69 to 6.58), age of acquisition (mean 38.74, SD 13.82, range 22.1 to 68.5), number of phonemes (mean 4.22, SD 1.60, range 1 to 11), number of syllables (mean 1.56, SD 0.73, range 1 to 5), percentage name agreement (mean 0.91, SD 0.12, range 0.3 to 1) and imageability (mean 594.96, SD 31.46, range 479 to 655). Additionally, no stimuli items within a set were first associates of each other, as determined by the Edinburgh Associative Thesaurus [53]. The auditory stimuli associated with pictures were spoken by a female voice and digitally recorded at 44100 Hz, mono, 32 bit, in a sound-proof recording studio.

### Procedure

**Figure 1:**
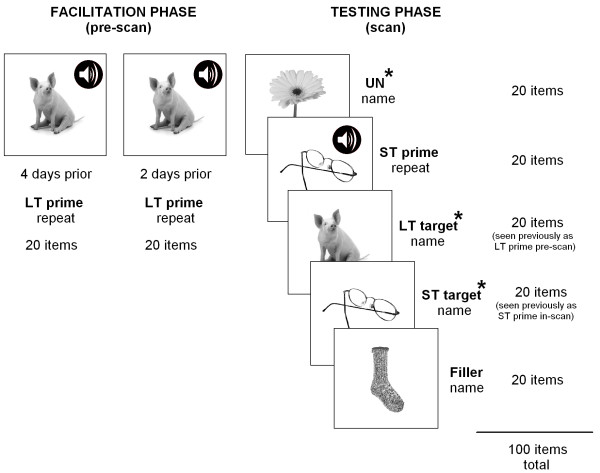
**A summary of the presentation of randomized stimuli**. *Facilitation phase*: one set of 20 pictures were presented three times on two separate occasions (six times total), simultaneously with their auditory names, for repetition (LT prime). *Testing phase (during scan)*: the long-term facilitated set were presented again for naming (LT target); a set of 20 pictures were presented twice - once as a prime along with the auditory name for repetition (ST prime) and then presented again (6 to 12 trials later) for naming (ST target); and one set of 20 unfacilitated pictures were also presented once for naming (UN), along with an additional set of 20 unfacilitated non-critical fillers. * indicates the three main naming conditions of interest - unfacilitated, long-term facilitated and short-term facilitated.

The study utilized a single independent factor of facilitation (short-term facilitated, long-term facilitated, or unfacilitated), with picture naming accuracy, reaction times and neural activity as measured by fMRI as primary dependent variables of interest. The entire experiment was conducted over the course of approximately two weeks and involved two main phases (see Figure 1). The first phase (facilitation phase) required each participant to complete two facilitation sessions, no more than three days apart, during which one set of 20 stimuli was presented three times, each time in a different random order. A single facilitation trial consisted of a fixation point (+) displayed for 1,500 ms, followed by display of a target picture for a period of 3,000 ms. Each target picture was presented simultaneously with its auditory name. Participants were required to overtly repeat the auditory name of each target item. Upon completion of both facilitation sessions, participants had been presented with each stimulus item from this one set of 20 items, along with its auditory name, a total of six times. Long-term facilitated items were presented repeatedly, due to the fact that word finding treatments are generally administered intensively and several repetitions of stimuli appear to be necessary to induce long lasting effects on naming ability [54]. No feedback was given regarding accuracy of responses within the facilitation sessions. The behavioral task utilized in the facilitation phase of the study was created using E-Prime (version 1.1) (Psychology Software Tools, Pittsburgh, PA).

The second phase (testing phase) was performed during an fMRI scanning session, with all three sets of stimulus items presented, plus an additional 20 items as non-critical fillers for naming. The behavioral task for the testing phase was created using Microsoft Visual Basic 6.0 (Microsoft Corporation, Redmond, WA). Naming responses were digitally recorded (sampling rate 11 kHz) with an optical single channel noise cancelling microphone (FOMRI, Optoacoustics Ltd., Or-Yehuda, Israel). The experimental stimuli were enlarged and back-projected onto the centre of a luminous white screen that the participants viewed through a mirror mounted on the head coil and subtended approximately 10° of visual arc. fMRI sessions were conducted in three runs, with two runs of 35 individual trials and one run of 30 trials, resulting in 100 trials in total. Each trial lasted 14.7 s and consisted of a 250 ms period of blank screen, followed by presentation of a target picture displayed for a period of 3 s. This was followed by a blank screen for 9.45 s, then a fixation point (+) displayed for 2 s to mark the commencement of the following trial. The long-term facilitation set of items, which were previously presented during the facilitation sessions, were presented again in the scanner to investigate any long-term facilitation effects (with no more than two days between the final facilitation session and scan). The short-term facilitation set of items were presented twice within the scanner (in different random order): once as a prime, along with the auditory name of that item, for overt repetition by participants and then presented again as a target (within a lag of 6 to 12 trials, average 10 trials) for naming to investigate any short-term facilitation effects (over a period of no more than 3 min). A set of unfacilitated items were also presented once within the scanner as a baseline for comparison purposes. Stimuli were presented pseudo-randomly in blocks of five trials per condition (long-term facilitated condition, short-term facilitated prime and target conditions, and unfacilitated condition), interspersed across the course of the scanning session. Stimuli were presented in blocks so that participants could be prepared for each type of task and thereby minimize any effects of constant task switching. In this regard, at the commencement of each trial block of five items, either the word "Name" (for main naming conditions of interest and filler items) or the word "Repeat" (for short-term facilitated prime items) was displayed in the centre of the screen to provide task instructions to participants.

### Image acquisition

Images were acquired using a 4-Tesla Bruker MedSpec whole body scanning system (Bruker Medical, Ettingen, Germany). The system utilized a transverse electromagnetic head coil [55] to enhance imaging resolution at a high field strength. Gradient-echo, echo planar images (GE-EPI) (matrix size of 64 × 64; repetition time (TR) 2100 ms; echo time (TE) 30 ms; 90° flip angle; field of view (FOV) 230 mm) with an interleaved gradient acquisition sequence were acquired in 36 axial planes with in-plane resolution of 3.6 mm and slice thickness of 3 mm (0.6 mm gap). To obtain minimal scanner noise during picture presentation and response time (4.2 s), a behavioral interleaved gradient design was employed, where only slice gradients were applied during the critical interval, with radiofrequency intact to maintain steady state magnetization [56]. For the following 10.5 s in which the blank screen (8.5 s) and fixation point (2 s) were displayed, image acquisition occurred to capture the blood oxygen level-dependent (BOLD) response for that naming trial. This design was primarily utilized to avoid artefacts associated with any head movement during an overt response. The design also allowed participants to hear auditorily presented stimuli, and permitted the recording of overt responses and accurate reaction times [56,57]. A total of 575 GE-EPI volumes were acquired over three runs, with the first five volumes (the first 10.5 s) in each run discarded to allow magnetization to reach steady state. A point-spread function (PSF) mapping sequence was acquired prior to GE-EPI acquisitions, allowing the distortion in geometry and intensity to be corrected in the time series data [58]. Within the same session a three-dimensional T_1 _weighted magnetization-prepared rapid gradient-echo (MP-RAGE) was acquired (matrix size of 256 × 256; TR 2200 ms; TE 2.99 ms; inversion time (TI) 900 ms; 9° flip angle; resolution 1 × 1 × 1 mm^3^; FOV 256 mm).

### Data processing

Incorrect responses and naming trials which elicited no response from participants (4.35% of responses), as well as data for the short-term facilitation primes, were treated as trials of no interest. Images were processed and analyzed using Statistical Parametric Mapping (Version 5) software (SPM5, Wellcome Department of Cognitive Neurology, London, UK) with MATLAB 2009a (The MathWorks Inc., Natick, MA). During spatial pre-processing the image time series were first realigned using rigid body motion correction with INRIAlign [59]. The mean EPI image generated from the realigned series for each participant was coregistered with the T_1 _image acquired in the same session. The T_1 _image was then segmented and normalized to the standard Montreal Neurological Institute (MNI) [60] atlas T_1 _weighted template. These transformations were applied to the realigned EPI time series. Normalized volumes (3 × 3 × 3 mm^3^) were then spatially smoothed using an 8 mm full-width half-maximum (FWHM) Gaussian kernel. Due to the partial collection of hemodynamic response function, a factor of the behavioral interleaved gradient design, a general linear model (GLM) for the fMRI time series was constructed using finite impulse response functions. Age was included as a covariate in the GLM and the onsets and durations were chosen to reflect the expected BOLD peak.

### Data analysis

Specific hypothesis-driven regions of interest (ROIs) were based upon the findings of various language-related meta-analyses, including Vigneau et al. [14]. In this regard, nine spherical ROIs (of 6 mm radius) in the left hemisphere were defined (MNI coordinates) using the MarsBaR region of interest toolbox [61] for SPM5 [62]. Three ROIs were identified as having been previously associated with phonological processing, including the posterior region of the inferior frontal gyrus (pars opercularis) (-54, 12, 20), the posterior portion of the superior temporal gyrus (-50, -38, 12) and the supramarginal gyrus of the parietal lobe (-42, -52, 37). Additionally, six ROIs associated with semantic processing were selected within the anterior (pars orbitalis) and mid (pars triangularis) portions of the inferior frontal gyrus (-37, 31, -9; -43, 20, 4), the anterior superior temporal gyrus (-56, -13, -5), the mid-section of the middle temporal gyrus (-59, -37, 1), the posterior inferior temporal gyrus (-46, -55,-7), and the angular gyrus of the parietal lobe (-45, -68, 26). It should be noted that the superior and middle temporal cortex ROIs chosen fall on the border of Indefrey and Levelt's [8] y coordinate delineation of middle (> -38) and posterior (< -38) temporal regions. Therefore, these two spherical ROIs may extend somewhat into the middle superior temporal region in the case of the posterior superior temporal gyrus and into the posterior middle temporal region for the mid-section of the middle temporal gyrus. A GLM, repeated measures analysis of variance (ANOVA) was conducted to compare main effects and corrected results are reported at *p *< 0.05 with false discovery rate (FDR) correction. Corrected *p*-values were calculated with the function "p.adjust" using the R statistical computing software package http://www.r-project.org/. Whole brain analyses were also conducted, with the neuroanatomical location of peak maxima identified using automated anatomical labelling software [63]. A height threshold of *p *< 0.001 was adopted in conjunction with a cluster threshold of *p *< 0.05 estimated for the whole brain (43 contiguous voxels) using a Monte Carlo estimation procedure with 10,000 simulations. The cluster threshold used as a variable in the Monte Carlo simulation (3dClustSim implemented in Analysis of Functional Neuroimages, National Institute of Mental Health, Bethesda, MD) [64] was determined by first calculating the FWHM of noise using the square root of the residuals (3dFWHMx).

## Results

### Behavioral results

**Figure 2:**
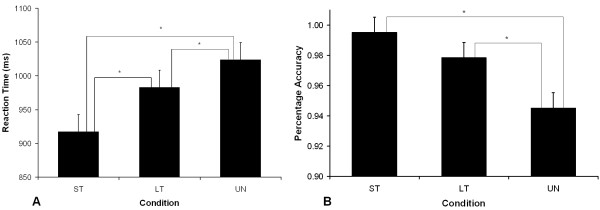
**Phonological facilitation effects in behavioral data**. Error bars indicate standard error mean. ***A***, Mean reaction times and standard errors for each condition. ***B***, Mean percentage accuracy for each condition. Significant differences were found between all conditions for naming latency (*p *< 0.001). A significant difference was also identified between the unfacilitated (UN) condition and both the short-term (ST) and long-term facilitated (LT) conditions for percentage accuracy (*p *< 0.001).

Naming latencies and accuracy data during the testing phase of the study are presented in Figure 2. An initial linear mixed model analysis, with subject as a random factor and condition as a fixed factor, was conducted on all behavioral data which included age as a covariate. The reaction time data analyses were conducted on correct responses, with times below 200 ms and above 1,500 ms removed (3.4% of correct responses) and the accuracy analyses conducted on all trials. Participants' age did not interact significantly with condition for either the reaction time (*F*_2,1153 _= 0.447, *p *= 0.640) or accuracy data (*F*_2,1254 _= 2.088, *p *= 0.124). A further analysis on the reaction time data showed a main effect for condition (*F*_2,1155 _= 55.431, *p *= < 0.001) and post-hoc pairwise comparisons identified significant differences between all conditions (*p *< 0.001) with both short- and long-term facilitated items named faster than unfacilitated items and short-term items faster than long-term facilitated items. A main effect of condition for accuracy was also identified (*F*_2,1257 _= 11.515, *p *< 0.001) with both short-term and long-term facilitated items named significantly more accurately than unfacilitated items (*p *< 0.001).

### Imaging results

**Figure 3:**
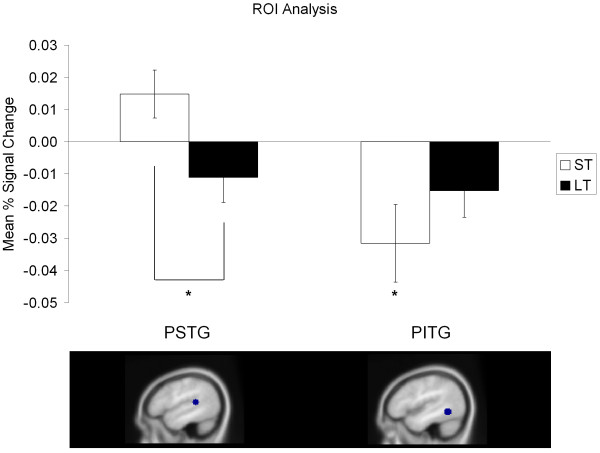
**Region of interest analysis**. Figure displays the a priori defined regions of interest within which significant differences in activation (*p *< 0.05) were identified for two key contrasts. Bar graph indicates relative mean percentage BOLD signal change as a function of facilitation, compared to the unfacilitated condition. PSTG = posterior superior temporal gyrus and PITG = posterior inferior temporal gyrus. * indicates significant changes in mean signal intensity (*p *< 0.05, FDR corrected) between conditions. Error bars indicate standard error mean.

**Figure 4:**
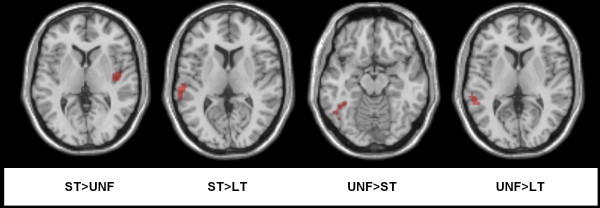
**Whole brain analyses**. Regions showing significant BOLD response for contrasts of interest (at *p *< 0.001) for clusters with a minimum of 43 contiguous voxels, displayed on the SPM5 T_1 _canonical brain in a series of axial slices.

Of the nine left hemisphere cortical ROIs examined, two showed significant differences in activation for key contrasts of interest following FDR correction (see Figure 3). Short-term facilitated items showed decreased activity in the posterior inferior temporal gyrus when compared to unfacilitated items (*p *= 0.041). In contrast, greater activation for short-term facilitated items than for long-term facilitated items (*p *= 0.027) was identified in the posterior superior temporal gyrus. Subsequent whole brain analyses (set out in Table 1 and Figure 4) revealed modulation of activation in comparable regions for the same two contrasts. Firstly, greater activation was found for short-term facilitated items within the left superior temporal gyrus when compared to long-term facilitated items. Secondly, involvement of the left inferior temporal gyrus was identified, with a decrease for short-term facilitated items relative to unfacilitated items. The whole brain analyses also identified a decrease in activation for long-term items when compared to unfacilitated items in the left middle temporal gyrus, and the right insula showed greater activation for short-term facilitated items than unfacilitated items. No significant activations that correlated with age were identified.

**Table 1:** MNI coordinates of peak activation.

Contrast Description and Anatomical Label	Volume	x	y	z	Z-score
Short-Term > Unfacilitated:					

right insula	82	45	-6	-3	4.43

Short-Term > Long-Term:					

left superior temporal gyrus	72	-63	-27	9	4.20

Unfacilitated > Short-Term:					

left inferior occipital gyrus extending to inferior temporal gyrus	43	-45	-69	-12	4.12

Unfacilitated > Long-Term:					

left middle temporal gyrus	45	-60	-42	9	4.49

Peak activation from the whole brain analyses (*p *< 0.001) for clusters with a minimum of 43 contiguous voxels

## Discussion

The aim of this study was to investigate the short- and long-term facilitation of overt picture naming using an auditory repetition task, in order to determine the neurocognitive substrates underlying facilitation from phonological tasks over time in healthy aging adults. It was hypothesized that short-term facilitation effects should engage regions associated with phonological processing, and that long-term facilitation may involve brain areas linked to both semantic and phonological processing. The present study found that long-term facilitation was indeed driven by modulation of activity in regions associated with both phonological (left posterior superior temporal gyrus) and semantic processing (left middle temporal gyrus). However, short-term facilitation was primarily associated with decreased activity in an area known to be involved in semantic processing and object recognition (left occipitotemporal region). Modulation of activity in areas associated with semantic processing may be due to the fact that both semantic and phonological processing occur to some degree with most language-related tasks [1]. In other words, although an auditory repetition task can be conceived as phonological, particularly when given as a treatment of word retrieval deficits in aphasia, it also involves semantic processing mechanisms. This is particularly the case when the picture is presented at the same time as the auditory word form and the speaker is assumed to understand the word being repeated.

The behavioral results (see Figure 2) showed that both short-term and long-term facilitated items were named significantly faster than unfacilitated items, with a larger priming effect for short-term as compared to long-term items. A similar pattern of priming was revealed in the accuracy data, with short-term and long-term facilitated items named significantly more accurately than unfacilitated items. The presence of these behavioral priming effects indicates that repeating auditorily presented stimuli can facilitate subsequent picture naming in healthy older adults and that the magnitude of this effect is reduced over time.

Neuroimaging results revealed decreased activity for facilitated conditions in both phonological and semantic regions, which could be attributed to "repetition suppression" - a relative decrease in cortical activity following repeated presentation of a stimulus, reflecting greater processing efficiency [30,47,65-68]. In addition, there was increased activity for short-term facilitated items in a neural region previously linked to phonological processing. This may reflect "repetition enhancement" - an increase in activity, which is often associated with additional processing upon repeated stimulus presentation [30,65]. We now turn to a discussion of findings, firstly in terms of neural regions where repetition suppression effects for facilitated items were identified and, secondly, areas where repetition enhancement effects for facilitated items were shown.

### Repetition suppression for facilitated items

Decreased activation for facilitated items was identified in three temporal regions: the superior, middle and inferior temporal gyri. This finding is largely consistent with previous picture naming studies investigating long lasting facilitation of naming, which have documented a decrease in activity for previously encountered items in occipitotemporal regions [30] and the posterior inferior temporal gyrus [31]. In the current investigation, both the ROI and whole brain analyses revealed a decrease in activity for long-term facilitated items relative to short-term facilitated items in the superior temporal gyrus, an area linked to phonological processing. More specifically, the superior temporal region is known to be involved in phonological access [13,33,69]. A repetition suppression effect here suggests that long-term facilitation may have resulted in more efficient activation and retrieval of phonological representations.

Both analyses also identified decreases in activation for short-term facilitated items relative to unfacilitated items in the inferior temporal region. The ROI analyses showed this decrease in activity within the posterior inferior temporal gyrus and the whole brain analyses identified a reduction in activity in the left inferior occipital gyrus, extending to the inferior temporal gyrus. In addition, the whole brain analyses identified a decrease in activation for long-term facilitated items when compared to unfacilitated items in the middle temporal gyrus. Both the middle and inferior temporal gyri are thought to be involved in lexical selection, where the lexical entry that matches a picture representation is accessed and selected [8,10,70,71]. Therefore, this decrease in activity for long-term facilitated items in the middle temporal region and for short-term facilitated items in the inferior temporal region, may be attributable to repeated and more efficient lexical selection of these previously presented items. It should be pointed out that, contrary to our original hypothesis regarding short-term effects primarily engaging regions associated with phonological processing, the most significant result within the ROI analyses was a decrease in activity for short-term facilitated items in the posterior inferior temporal gyrus (refer to Figure 3). As previously noted, although our facilitation task attempted to focus on phonological processing, it could be the obligatory semantic aspects of the task (i.e. presence of the picture and/or understanding the word repeated) that play a major role in driving facilitatory effects. Additionally, object priming studies commonly report decreases in activation within occipitotemporal regions [30,31,46,72]. A decrease for short-term facilitated items in the inferior temporal gyrus, therefore, may also represent more efficient object recognition, or indeed shape recognition, which is considered to be the most salient feature associated with recognition of an object [73]. It is notable that although our long-term facilitated items were seen a total of six times prior to scanning, recognition priming effects were identified in areas linked to visual object recognition only for short-term facilitated items. It appears that recognition priming effects were influenced by recency more than dosage in the current study.

In summary, a repetition suppression effect for long-term facilitated items was identified in regions associated with both phonological and semantic processing, supporting our hypotheses and the view that a strengthening of semantic-phonological connections may be associated with longer lasting facilitation. An auditory repetition task in the presence of the picture appears to have resulted in more efficient activation and selection of both semantic and phonological representations. In contrast to our hypotheses, however, for short-term facilitated items a repetition suppression effect was found only within the inferior occipitotemporal region, suggesting that short-term facilitation of naming was primarily driven by semantic processing, as well as object recognition processes.

### Repetition enhancement for facilitated items

The facilitatory effects related to tasks based on priming are generally associated with a relative decrease in neural activity due to greater processing efficiency [30,72]. In light of our behavioral results, which showed faster and more accurate responses for facilitated items, we would expect more efficient processing and a generalized decrease in neural activity on subsequent naming. For long-term facilitated items relative to short-term facilitated items this was the case, with a repetition suppression effect identified in the left superior temporal gyrus. However, although this contrast has already been interpreted with regard to a repetition suppression effect for long-term items, it can also be accounted for in terms of a repetition enhancement effect for short-term facilitated items. It is possible that both repetition suppression and enhancement effects in this region are contributing to facilitation and we are unable to lend more support for either interpretation based on the current findings. Therefore, we now also discuss this result in terms of an increase in activity within the superior temporal gyrus for short-term facilitated items.

Other functional neuroimaging studies based on repetition priming have also found increased activity in the superior temporal region, in some cases for repeated, rather than novel stimuli with parallel reductions in response latencies [67,72,74] and have argued that this may be indicative of automatic recognition incidental to task requirements. The primary auditory cortex forms part of the superior temporal gyrus and this cortical region is known to be involved in speech perception, as well as phonological access in general [13,33,69]. In fact, this region has been linked to phonological level processing in both speech perception and speech production [75-77] and word production studies requiring overt responses consistently report bilateral superior temporal region involvement [8]. It appears that the posterior superior temporal gyrus represents an important anatomical site for a certain degree of functional overlap for phonological processing [74]. With this in mind, we put forward a speculative explanation for a repetition enhancement effect in the superior temporal region.

An increase in neural activity to stimulus presentation is often reported where additional cognitive processing is required [30,65]. As the task required during each of our three conditions of interest was overt naming, theoretically there should not be any additional processing required for short-term facilitated items. Therefore, enhanced activity restricted to short-term facilitated items in the superior temporal gyrus may involve active recall or recognition of the auditory prime presentation and overt repetition of the word form performed a short time previously. This heightened recall of the phonological word form could result in an increase in neural activity, with an associated reduction in reaction times due to more efficient phonological processing during subsequent naming. This relationship between neural activity and response latencies would not be expected for unfacilitated items, and in the case of long-term facilitated items, may not be as pronounced. An active recall mechanism operating at an area of partial overlap for phonological perception and production systems may be responsible for the increased activity observed for short-term facilitated items in the superior temporal gyrus. Additionally, evidence linking this region to phonological processes involved in verbal short-term memory provides support for this suggestion [70,74,75].

Finally, the whole brain results identified greater activation for short-term facilitated than unfacilitated items in the right insula. The left insula region has been linked to the planning and coordination of overt speech articulation [78,79]. Previous studies have also identified an increase in activity in the left insula thought to contribute to a learning-related change in the phonetic representation of a picture, as naming becomes more automatic [30,46]. Restriction of activity to the right insula region in the present study cannot be explained immediately, but some research suggests involvement of this region in relevant processes. Mechelli et al. [29] manipulated the phonological and semantic relationship between successive pairs of stimuli and found that phonologically related pairs of both written words and pictures modulated neural activity in bilateral insula regions. Further, a review by Ackermann and Riecker [80] suggests that the right insula contributes to prosodic aspects of verbal utterances and melodies.

This study did not aim to examine age-related changes in processing, however, effects of age on priming were examined in order to exclude ageing as a potential confound. Previous research has shown an age-related decline in picture naming ability [81,82], associated with activity changes in cortical regions subserving language tasks [83,84]. The literature investigating aging and priming effects, however, is less extensive and while early behavioral studies found a reduction in priming effects in very elderly participants [85,86], more recent research argues that priming is age-invariant [87-89]. In line with these more recent findings, the present study found no age-related priming effects in this healthy older cohort. Despite this, it should be noted that the results of the current study may not be generalizable to the wider population and comparison to other studies may be limited due to differences in age of participants.

As previously mentioned, the type of task employed in the current study is similar to those used in clinical treatments of word retrieval deficits in aphasia. Consideration of the mechanisms underlying the facilitation of unimpaired picture naming has the potential to inform the therapeutic facilitation of naming in the recovery and reorganization of word production abilities following acquired neurological injury. At present any neuroplasticity associated with the treatment of word retrieval in aphasia is considered in the context of brain mechanisms engaged during standard picture naming, but not in terms of normal priming mechanisms which may be more relevant to naming treatment effects. In this regard, further research should explore the effects of a variety of facilitation techniques in unimpaired speakers, and investigate how these effects can be utilized in the treatment of word production disorders in neurological populations.

## Conclusions

These results suggest that different neurocognitive mechanisms underlie short- and long-term facilitation of naming with an auditory repetition task. Our findings add to the debate stemming from behavioral studies as to whether facilitation from phonological tasks is occurring at the level of word form representation, or through strengthening the connections between semantic and phonological levels of processing. It appears that in the long-term, facilitation of subsequent naming elicits repetition suppression effects in regions associated with both semantic and phonological processing, reflecting a strengthening of semantic-phonological connections. Finally, short-term facilitation effects appear to be driven primarily by the semantic or object-based aspects of the task, with repetition suppression effects restricted to a region associated more strongly with semantics and object recognition. This short-lived facilitation may be contributed to by a repetition enhancement effect in a phonological region, possibly associated with active recognition of the phonological form.

## Authors' contributions

SH contributed to study design, performed behavioral and neuroimaging testing, conducted data analyses and interpretation, and wrote the manuscript. KM contributed to the design of the study, oversaw neuroimaging data collection, contributed to analyses and interpretation, and revised the manuscript. LN contributed to study design, assisted with interpretation of results and revised the manuscript critically for intellectual content. AA helped to design the study and revise the manuscript for intellectual content. AM contributed to study design, assisted with data collection and analysis, as well as helped to revise the manuscript. SvH and KJ were involved with data collection and analysis. DC was responsible for the conceptualization and design of the study, contributed to statistical analyses, assisted with interpretation of results and revised the manuscript critically for important intellectual content. All authors read and approved the final manuscript.
